# Phylogeography of Rift Valley Fever Virus in Africa Reveals Multiple Introductions in Senegal and Mauritania

**DOI:** 10.1371/journal.pone.0035216

**Published:** 2012-04-23

**Authors:** P. O. Ly Soumaré, Caio C. M. Freire, Ousmane Faye, Mawlouth Diallo, Juliana Velasco C. de Oliveira, Paolo M. A. Zanotto, Amadou Alpha Sall

**Affiliations:** 1 Institut Pasteur de Dakar, Dakar, Senegal; 2 Laboratory of Molecular Evolution and Bioinformatics, Department of Microbiology, Biomedical Sciences Institute, University of Sao Paulo, Sao Paulo, Brazil; The University of Texas Medical Branch, United States of America

## Abstract

Rift Valley Fever (RVF) virus (Family Bunyaviridae) is an arthropod-borne RNA virus that infects primarily domestic ruminants and occasionally humans. RVF epizootics are characterized by numerous abortions and mortality among young animals. In humans, the illness is usually characterized by a mild self-limited febrile illness, which could progress to more serious complications. RVF virus is widespread and endemic in many regions of Africa. In Western Africa, several outbreaks have been reported since 1987 when the first major one occurred at the frontier of Senegal and Mauritania. Aiming to evaluate the spreading and molecular epidemiology in these countries, RVFV isolates from 1944 to 2008 obtained from 18 localities in Senegal and Mauritania and 15 other countries were investigated. Our results suggest that a more intense viral activity possibly took place during the last century compared to the recent past and that at least 5 introductions of RVFV took place in Senegal and Mauritania from distant African regions. Moreover, Barkedji in Senegal was possibly a hub associated with the three distinct entries of RVFV in West Africa.

## Introduction

Rift Valley fever (RVF) is a mosquito borne anthropozoonosis affecting livestock and also humans throughout Africa and the Arabian Peninsula. Animal infections lead to high mortality among young ruminants and abortions of pregnant females while human infections are associated with a wide array of syndromes ranging from influenza-like illness to severe symptoms including hemorrhages, encephalitis, hepatitis, ocular complications and fatal outcomes [1]. Animal and human mortality and morbidity caused by RVF often result in high public health burden and substantial economic losses in endemic countries [2]. RVF is caused by the RVF virus (RVFV), a phlebovirus (Family Bunyaviridae) with three single-stranded RNA segments genome: L (large), M (medium) and S (small). The L segment encodes the RNA-dependent RNA polymerase [3], the M segment envelope glycoproteins G1 and G2 and two non-structural proteins of 14 and 78 kDa molecular weight, respectively [4]. The S segment has an ambisense coding strategy, since it encodes for the nucleocapsid protein (N) on the 5′-3′, positive sense and a non-structural protein (NSs) in the 5′-3′, negative sense [5]. The latter protein plays a major role in innate immunity by blocking interferon gene expression [6].

Although RVF was discovered in 1930 [7], [8], the disease remained a veterinary concern until a major outbreak occurred in Egypt in 1977 [1], [9], where RVF caused about one million human infections, 600 deaths and severe widespread epizootics [10]. Subsequently, more studies on RVFV transmission characterized additional major outbreaks derived from endemic sub-Saharan African countries [11]–[16]. The exchange from the enzootic cycle (*i.e.*, among wild animals) to peridomestic transmission (*i.e.*, among animals such as cattle, lambs and goats) is thought to cause the occasional spillover outbreaks into humans [17]. The most recent epidemics occurred in Kenya, Somalia, Tanzania in 2007 [18], South Africa in 2008 [19] and again in 2010 [20], Sudan in 2008 where more than 200 deaths were reported [21] and Mauritania in 2010 (Faye, unpublished data). A second introduction occurred when RVF, coming from East Africa, moved into Saudi Arabia and Yemen in the Arabian Peninsula [22]. It went for the first time outside of Africa –where it had been confined so far– becoming a threat to the Middle East. In the light of its natural history, RVFV appears to be a great example of an emerging pathogen with great potential of dispersion and impact on human and animal health and, a good model for an integrated approach to human and animal health (*i.e.*, the ‘one health’ concept). Several authors using molecular phylogeny methods attempted to unravel mechanisms underlying its distribution and dispersal in Africa [16], [23]–[26]. These studies revealed that: (*i*) RVFV genomes carry low genetic diversity ranging from 1 to 5% sequence divergence, (*ii*) change at a mean evolutionary rates ranging from 2.8 to 3.9E-4 substitution/site/year [27], (*iii*) can be grouped into several lineages [23], [25], (*iv*) can be grouped by geographic origin [16], (*v*) undergo reassortment in nature [28] and, (*vi*) the extant genetic diversity coalesces about 120–130 years ago [23]. However, except for Kenya [27], limited information is available on the evolution of RVFV in a country or regional scale. This lack of information poses the necessity to investigate the determinants of RVFV circulation in West Africa. In order to fill this gap, we sequenced 48 RVFV isolates from Senegal and Mauritania and along with sequences from Guinea and Burkina Faso, to allow inferences on the dispersal patterns of RVFV in West Africa. We also added to the study samples from East Africa to establish migratory patterns in Africa at a coarse grain.

## Results and Discussion

### Sequence analyses

Forty-eight RVFV isolates collected over a period of 20 years (1983 to 2003) from different areas of Senegal and Mauritania were included in the study (Table S1). For each strain NSs, G2, and polymerase genes were partially sequenced. Overall levels of sequence diversity and polymorphisms were within ranges previously observed and no deletions or insertions were found [24], [29]. Our analyses of recombination, using several different methods, showed the absence of intra gene recombination on the regions we studied and low levels of genetic diversity, in agreement with what was previously observed by [16], [23]–[27]. There are many plausible explanations for the low rates of genetic change estimated for RVFV, which could entail factors involved in genetic variability reduction, ranging from speed of replication, high rate of vertical transmission to reduced viremia. Moreover, the alternation between vertebrates and invertebrate hosts during the RVFV life cycle could hinder viral variation by imposing strong selective advantage in keeping highly adapted phenotypes [30]. Likewise, selection analyzes of the three genomic segments uncovered several sites under strong negative selection, indicated by ω<0, (Table 1) shows purging of deleterious polymorphisms in functionally important genes. On the other hand, a lack of positively selected sites indicated by ω>0, shows a lack directional change, typical of highly adapted genotypes. These findings also agree with the fact that the natural cycle of RVFV imposes several transmission bottlenecks due to alternation between a diverse set of arthropods vectors and mammal hosts that keep selective pressure towards genomic stability, such as the preservation of the S segment *in vivo* [30]. Moreover, the genomic stability we observed is consistent with the enzootic status of RVFV in several places in Africa.

**Table 1:** Negatively selected codon sites detected in RVFV.

Genomic segment	Amino acid position*	ω¶	*p*-value
Large	559	−5.0648	0.0085
Large	860	−5.0648	0.0085
Large	956	−5.0648	0.0085
Large	1091	−6.3310	0.0030
Large	1332	−5.0648	0.0085
Large	1870	−5.0648	0.0085
Medium	64	−5.1841	0.0093
Medium	172	−54.8731	0.0072
Medium	225	−6.9101	0.0021
Medium	282	−4.5937	0.0071
Medium	439	−5.0000	0.0041
Medium	971	−5.5281	0.0071
Medium	1115	−11.6919	0.0047
Small	116	−5.0000	0.0047
Small	124	−4.5667	0.0073
Small	127	−8.0000	0.0002
Small	148	−4.5667	0.0073
Small	151	−5.9157	0.0048

* The amino acid position was related to first codon of sequence from Mauritanian OS8 strain. The accession numbers of Large, Medium and Small segments are, respectively, DQ375395.1, DQ380185.1 and DQ380177.1.

¶ Negatively selected sites (ω<0) identified by the significant difference between non-synonymous (*dN*) rates and synonymous (*dS*) rates per site using the Single Likelihood Ancestor Counting (SLAC) method with HyPhy.

### Phylodynamics of RVFV

**Figure 1:**
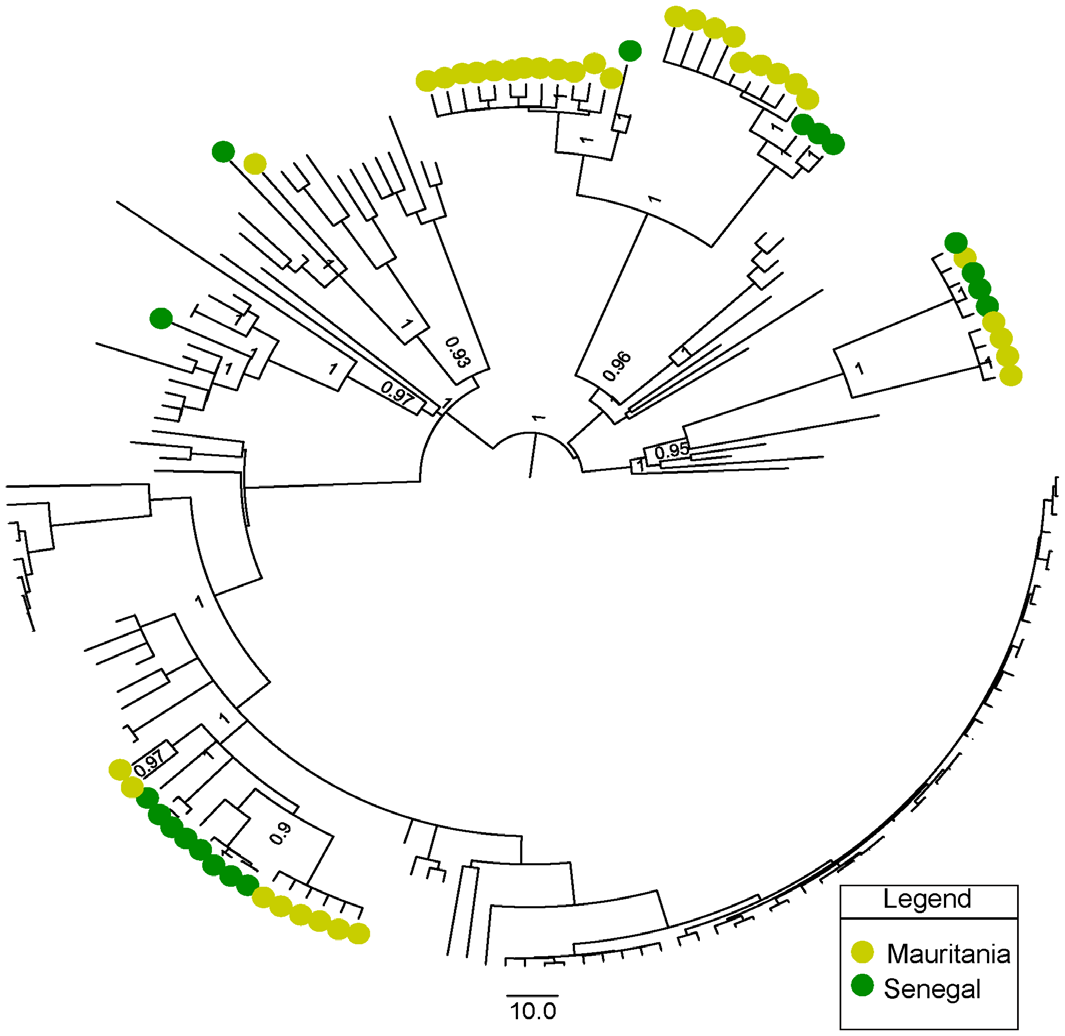
Maximum clade credibility (MCC) tree for the short (S) segment. Relationship among 167 strains of RVFV isolated from different localities and countries. Samples from Mauritania and Senegal are shown by green and yellow dots respectively. The recurrent independent clustering of green and yellow dots suggests multiple introductions of RVFV into these countries throughout the 20^th^ century. Posterior probability values greater than 90% are shown near tree nodes.

**Figure 2:**
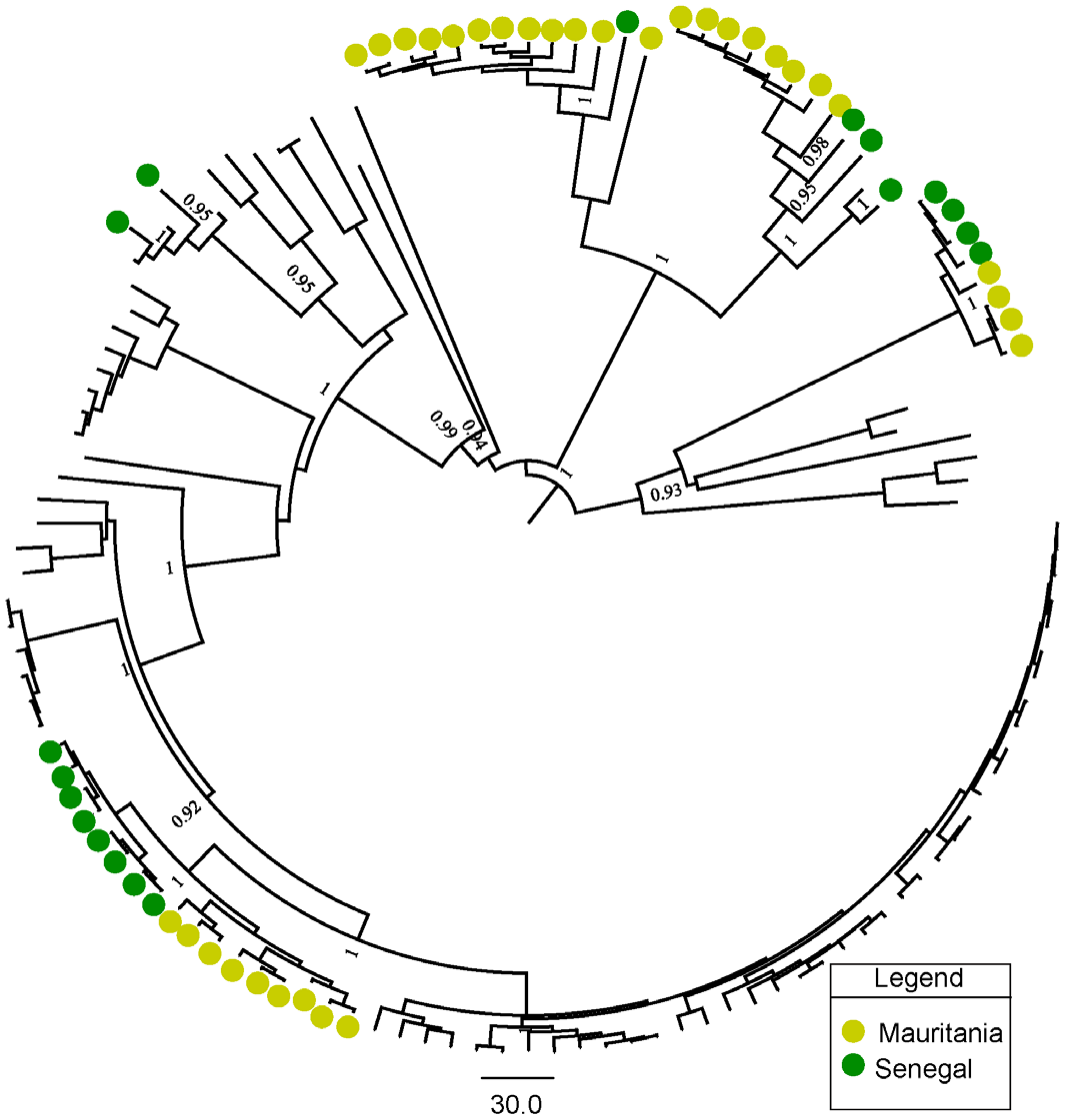
Maximum clade credibility (MCC) tree for the medium (M) segment. Relationship among 128 strains of RVFV isolated from different localities and countries. Samples from Mauritania and Senegal are shown by green and yellow dots respectively. The recurrent independent clustering of green and yellow dots suggests multiple introductions of RVFV into these countries throughout the 20^th^ century. Posterior probability values greater than 90% are shown near tree nodes.

**Figure 3:**
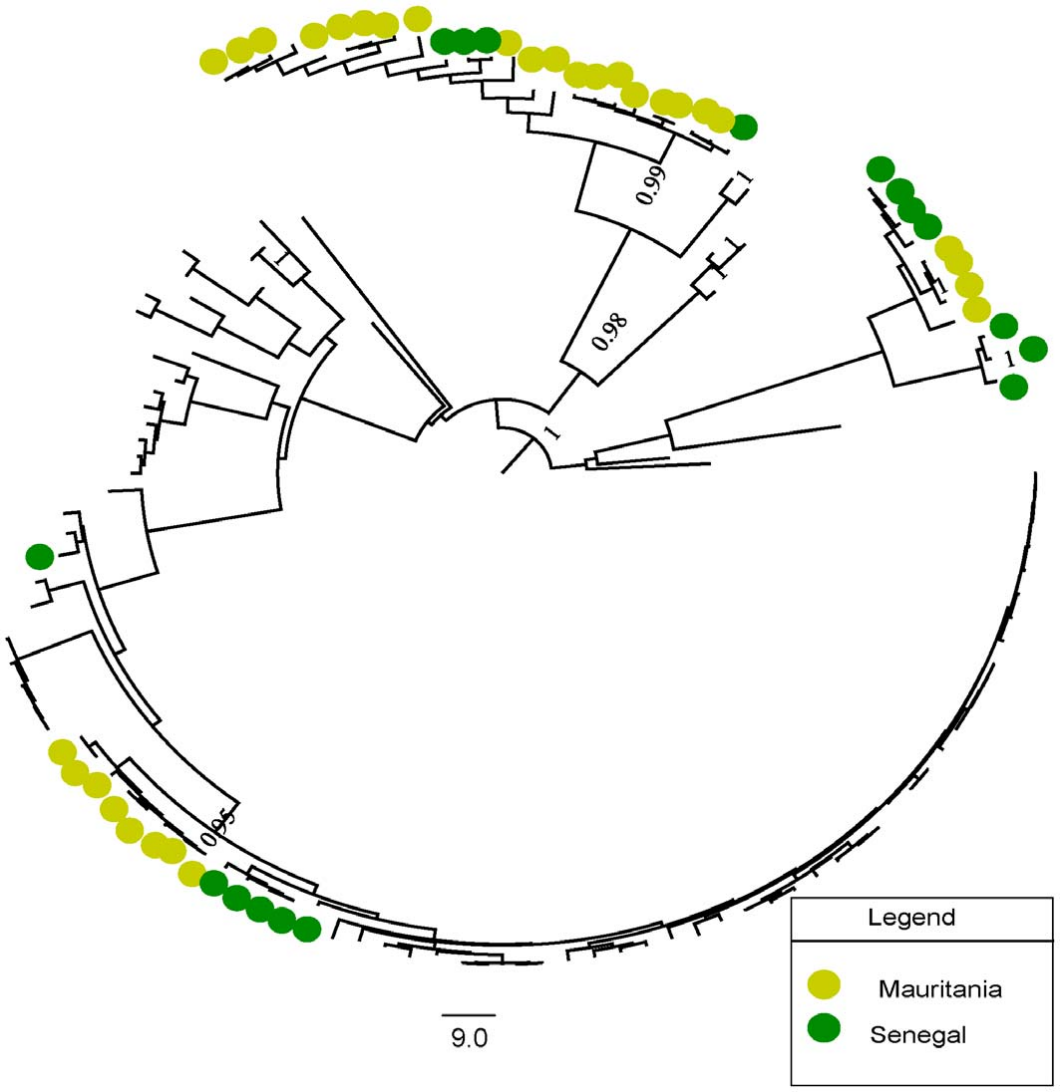
Maximum clade credibility (MCC) tree for the long (L) segment. Relationship among 126 strains of RVFV isolated from different localities and countries. Samples from Mauritania and Senegal are shown by green and yellow dots respectively. The recurrent independent clustering of green and yellow dots suggests multiple introductions of RVFV into these countries throughout the 20^th^ century. Posterior probability values greater than 90% are shown near tree nodes.

We first investigated the phylogenetic signal content in our data by reconstructing 50 thousand quartets for each gene segment using the likelihood mapping method (see methods section). Our results (Table S2) indicated that the M segment had the higher phylogenetic signal content given its lower percentage of unresolved quartets, followed by the L and S segments. Maximum clade credibility (MCC) trees obtained during Bayesian inference for the S (Figure 1), M (Figure 2) and L (Figure 3) segments and trees obtained by maximum likelihood (data not shown), grouped samples from Senegal and Mauritania as sister taxa, as indicated by the preferential clustering of yellow and green dots in the tips of distinct clades in the trees. The occurrence of green and yellow dots in five distinct groups of taxa in Figure 1, 2 and 3 is evidence for independent shared introductions in these countries. These findings were well supported by significant posterior probabilities and by high bootstrap values for maximum likelihood trees, inferred with GARLI (data not shown). Furthermore, the phylogenetic adjacency of lineages, indicated by clustering of green and yellow dots in Figure 1, 2 and 3, indicated a recurrent exchange between the two neighbor countries during the last century. We then did phylogeographic reconstructions based on the S segment alone, since it had the highest posterior probabilities during state reconstructions. This allowed a coarse-grained view of the spatio-temporal patterns of RVFV spread in the African continent in general and, the pattern of movement into Mauritania and Senegal in particular. Importantly, the time to the most recent common ancestor (TMRCA) parameter values that we estimated from sequences from each genetic segment were different. This could be explained to some extent by the large and distinct number of negatively selected sites among segments, which could lead to an underestimation of the age of nodes near to the root of tree [31]. In addition, albeit obtaining high posterior values in MCC trees, we used different-sized sequences during our reconstructions of viral genealogies, which is acceptable within the likelihood framework, but could nonetheless, lead to underestimation of branch lengths for taxa with shorter sequences [32]. On the other hand, segmented genome allows reassortment events [28] that even at low frequency [23], this could cause differences in TMRCA estimates for different segments.

**Figure 4:**
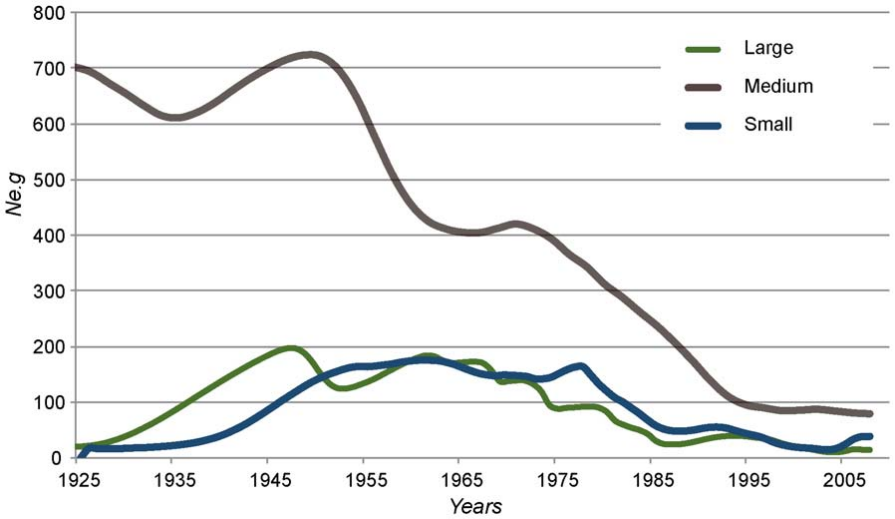
Bayesian skyride plot (BSP) for the three genomic segments of RVFV. The effective population size times the generation time (*Ne.g*) parameter approximates the number of infections in time. The plots overlay indicated complex oscillations with higher viral activity starting at around 1930, culminating sometime between the 40's and 60's, followed by a steady decrease from the 70's to the present. The stabilization of near the present is consistent with enzooticism, since the virus is not kept in human populations.

Based on the S segment of the RVFV that better resolved phylogeographic patterns, we observed that our trees had temporal coherence, since the age of key nodes of the S MCC tree agreed with known events, such as the first report of RVFV in Kenya in 1930 [7], [8] and the estimated arrival in this country from Zimbabwe. A root for the tree was dated between 1909 and 1920 and placed somewhere near the Southern tip of the Rift Valley. We assume that variation in genetic diversity in time (measured as effective population size times the generation time, *Ne.g*) estimated with the Monte Carlo Markov Chain (MCMC) method, correlates with RVFV demography and is proportional to the number of infections in time. The Bayesian skyride plots (BSP) for all genomic segments of RVFV had an increase in *Ne.g* in the first half of last century followed by a continuous decrease in the last half (Figure 4). Moreover, the BSPs agreed with a recent origin of RVFV and intense viral activity in the first half of 20^th^ century followed by dispersal in the African Continent. While the reduction in viral population size or infection events (*Ne.g*) are consistent with enzootic activity near the present. This finding agrees with the RVFV spread among large populations of susceptible hosts following to the introduction of livestock in East Africa namely Kenya in the first half of the 20^th^ century to improve husbandry [27], [33]. In essence, the higher levels of *Ne.g* in the past (Figure 4) agree with the fact that RVFV has been active over the last century causing major outbreaks and suggest a recent trend of enzootism.

### Phylogeography of RVFV in the African continent

**Figure 5:**
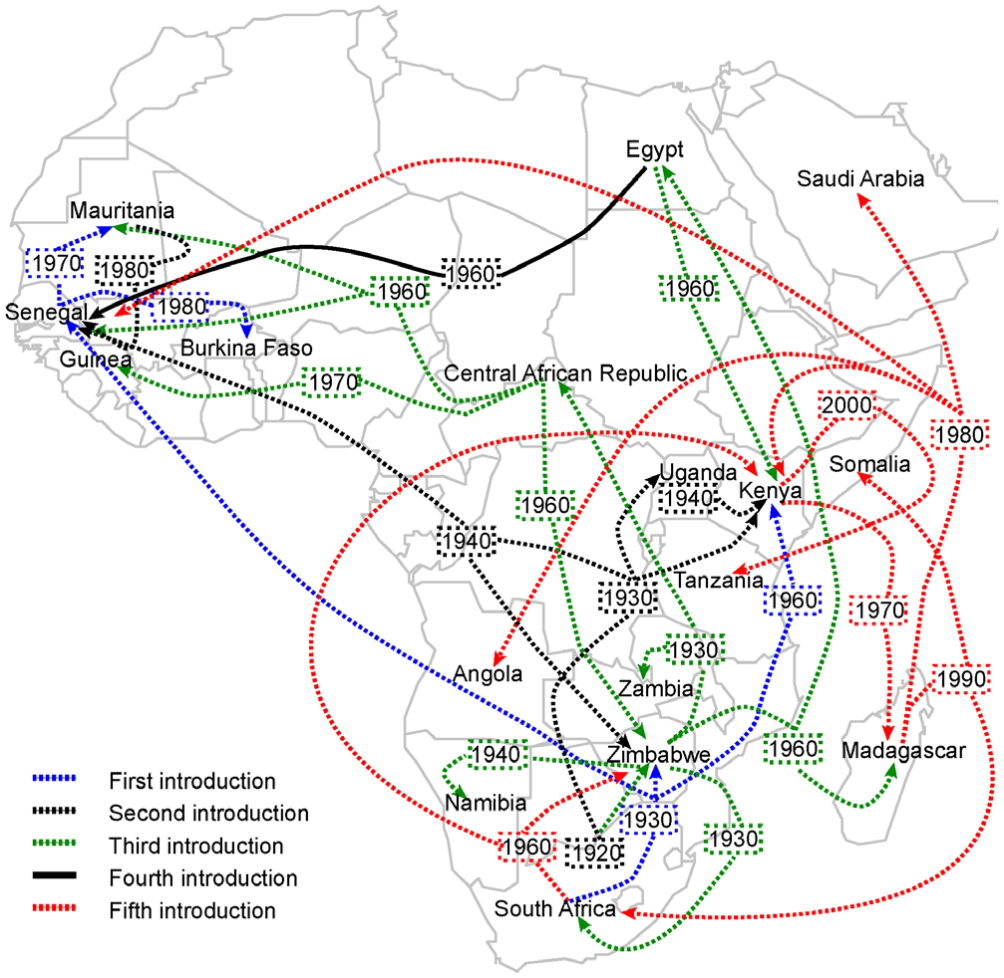
Large-scale geographic spread of RVFV in Africa and Saudi Arabia based on the S segment. The directed lines connect the sources and target localities (shown by arrows) of viral lineages. The distinct introductions into Senegal and Mauritania were represented by different colors. The estimated time to the most recent common ancestor of strains from different countries are shown within rectangles and could be interpreted as the oldest possible year of introduction of that lineage at that locality.

**Figure 6:**
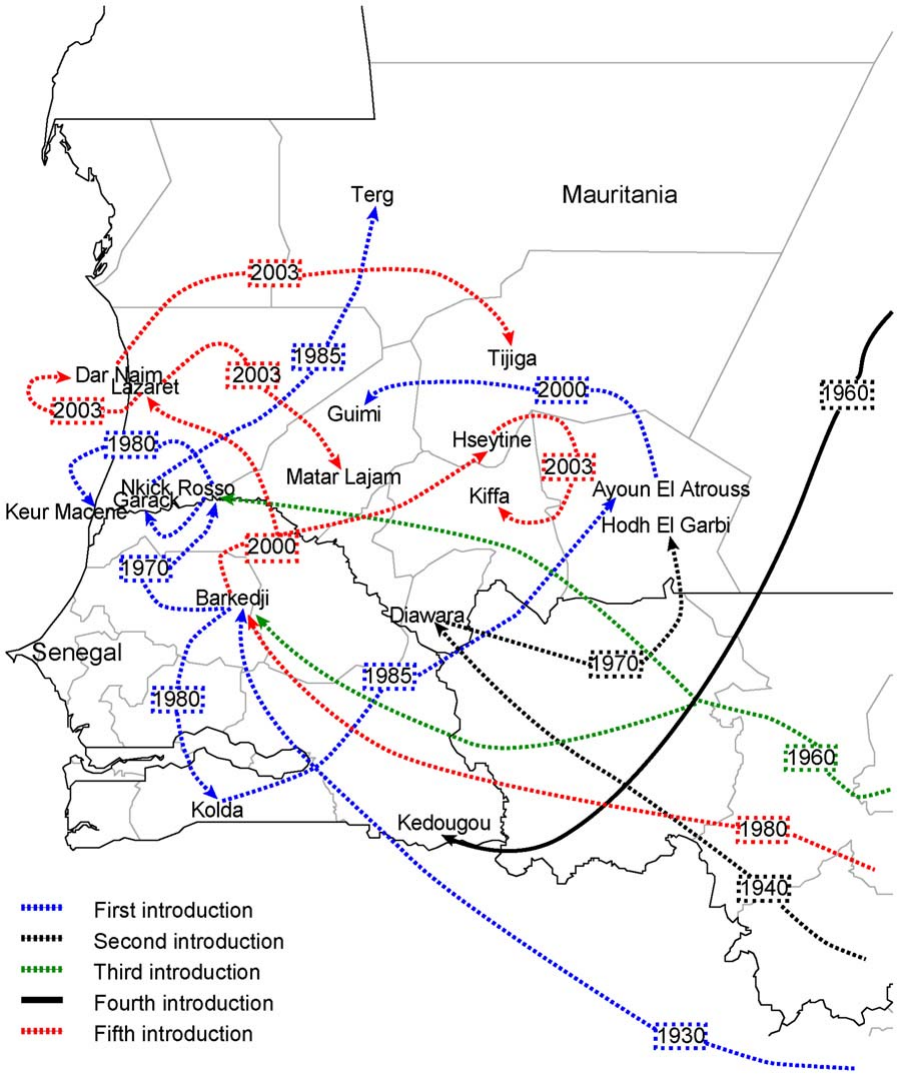
Geographic spread of RVFV in Mauritania and Senegal. The directed lines connect the sources and target localities (shown by arrows) of viral lineages. The distinct introductions into Senegal and Mauritania were represented by different colors. The estimated time to the most recent common ancestor of strains from different countries are shown within rectangles and could be interpreted as the oldest possible year of introduction of that lineage at that locality.

Phylogeographic reconstructions summarized in Figure S1 unveiled a complex pattern of viral movement across long distances in the African continent that we represented in maps (Figures 5 and 6). The dates in the map arrows in Figures 5 and 6 indicate the reconstructed TMRCA for a lineage found at the locality shown by the arrowhead and is assumed as the oldest possible year of introduction of that lineage at a given locality. We can observe a stepwise spread from a locality to the next, by focusing on East Africa, were denser sampling allows greater detail (Figure 6). Our inferences unveiled five distinct introductions in Senegal and Mauritania during the past century (Figure 5 and KML file in Dataset S1). The first arrival in West Africa (blue line in Figure 5) was related to strains from Zimbabwe, South Africa and Uganda possibly from around 80 years ago, spreading to several locations in Senegal (SN) and Mauritania (MR). These inferences are consistent with serological surveys done by the Pasteur Institute in Dakar that suggest that the coastal zone of West Africa may have had RVFV during the first half of the last century (data not published). The second introduction to West Africa (black line in Figure 5 and Figure 6) was associated with strains from South Africa or Zimbabwe sharing a common ancestor around 72 years ago. This introduction was associated with samples from Diawara (SN) and Hodh El Garbi (MR), and an outbreak in 1998 in Diawara [15]. The third introduction in West Africa (shown as a green line in Figure 5 and Figure 6) consisted of strains that originated in Zimbabwe in the beginning of last century, then moved to Central African Republic around 70 years ago and, from there reached Senegal and Mauritania around 47 years ago and Guinea 38 years ago. Samples from this introduction were isolated in Barkedji (SN) in 1993 and Rosso (MR) in 1987 and in Guinea in 1981 and 1984. The fourth introduction (shown as solid black line in Figure 5 and Figure 6) was evidenced by a single strain isolated in Kedougou in 1983 associated with an old South African lineage that moved to Egypt samples around 47 years ago and Madagascar around 40 years ago. The most recent introduction in west Africa (fifth introduction, red line, in Figure 5 and Figure 6) was due to a wave of RVFV that spread to distant places in Africa caused by a lineage from the Southern part of Africa that moved to Zimbabwe and Kenya 47 years ago and then reached Madagascar, Somalia, Tanzania, Angola, Senegal, Mauritania and Saudi Arabia during the last 28 years. The most interesting feature of this RVFV wave was that it recurrently reemerged in Kenya from where it was broadcasted to other localities.

### Phylogeography of RVFV in West Africa

The history of introductions, exchanges and spread of RVFV among Senegalese and Mauritanian territories is shown in greater detail in Figure 6. The map in Figure 6 summarizes independent phylogeographic reconstructions, one for each introduction. The MCC trees (available from authors upon request) agreed well with global MCC tree Figure S1. According to our reconstructions, the first introduction possibly originating from South Africa in the 30's appears to have taken place in Barkedji (SN). Subsequently, it moved to Rosso (MR) around 1970 and Kolda (SN) around 1980. The Rosso lineage was then broadcasted to the provinces of Garack, Nkick and Keur Macene in Mauritania around 1980. Later in 1985, a lineage from Nkick moved into Terg in the North of Mauritania. On the other hand, a lineage from Kolda moved into east Mauritania to Ayoun El Atrouss around the end of the 80's, and from there, to Guimi around 2000. The second introduction, possibly from central Africa, reached Diawara in Senegal around 1940. From there it moved to Hodh el Garbi in Mauritania around 1970. As for the first and third introductions, the fifth (most recent introduction, indicated by a red line) also appears to have entered Senegal in Barkedji and from there, reached the Mauritanian territories of Lazaret and Hseytine. They then spread from Hseytine to Kiffa (MR) and from Lazaret to Matar Lajam and Dar Naim and from the last to Tijiga during the last decade.

### Biologic correlates of complex RVFV spread patterns

By contrasting different scales of RVFV spread, fine-grain in West Africa and coarse-grained elsewhere in the continent, we unveiled an intricate dispersal pattern that shows: (*i*) viral dispersal across long distances, some times at fast rates, as indicated by the large distances among places of isolation of closely-related strains [26], (*ii*) persistence in different places for more than 70 years, like that observed for old lineages from South Africa, Zimbabwe, Central African Republic, Kenya and Senegal and, (*iii*) a gradual spread at nearby localities that we show in greater detail in Senegal and Mauritania. The capacity of RVFV to cross large geographical extensions, could be assisted by the movement of infected mammals and mosquitoes [34]. Likewise, animal herds, acting as reservoirs, could warrant the maintenance of RVFV enzootic circulation at a regional level [35]. The spread of RVF from endemic regions to areas without disease through animal migration routes has been postulated for previous outbreaks. For example, RVFV has been exported to Egypt in 1977, probably by lambs from Sudan [36] and from Eastern Africa to Madagascar in 1991 [16] and then to the Arabian Peninsula in 2000 [37]. Nevertheless, some evidence for enzootic activity has also been put forward. For example, it has been proposed that there is persistent enzootic activity of RVFV in Senegal based on seroprevalence data, which could help explain outbreaks in Mauritania and Egypt without heavy rain falls [13]. Moreover, an enzootic cycle has been described in Barkedji, which could be maintained by vertical transmission among *Aedes* mosquitoes [27].

Crucially, our results also support the notion that Barkedji functions as a hub, broadcasting RVFV to other localities in West Africa. At a coarse grain, we observed that the Zimbabwe spread viruses during the first half of last century, while Kenya experienced a more intense activity as a hub during the second half. The notion that Barkedji may be an important gateway to RVFV in Senegal and Mauritania was previously suggested by serologic and entomologic surveys [13], [14]. An important role of Barkedji appears to be independently supported by the fact that it is a known crossroad of migration movements of herds between the southern and northern regions of Senegal and, to a larger extent, to southern Mauritania. Moreover, the distinct introductions in Barkedji may help explain the maintenance of the endemic cycle at the regional scale, by feeding the zoonotic pool required for its persistence [35]. Because mosquitoes cannot fly more than a few hundred meters during their lifetime, their role in long-range dissemination may be limited [38]. This important role however, could be fulfilled by infected members of roaming herds that could help the introduction and dispersion of RVFV strains across Africa [39]. Perhaps the lack of well-characterized reservoirs in both sides of the Senegalese Valley could be explained by a constant replenishment of potential hosts in the area. Our data agrees with the notion that infected hosts may regularly introduce and reintroduce viruses through places such as Barkedji, possibly through migratory routes. Nevertheless, any additional understanding of the putative role of Barkedji as a RVFV gateway to Senegal and Mauritania requires investigating a multitude of factors and processes that ultimately would help maintain and replenish the enzootic pool.

## Materials and Methods

### Ethics Statements and Clinical samples

The study was submitted and approved by the Ethics Committee of Institute Pasteur of Dakar in Senegal. Samples from Senegal and Mauritania are part of the Institute Pasteur in Dakar CRORA collection (Centre collaborateur OMS de référence et de recherche pour les arbovirus et virus de fièvres hémorragiques). Animal and human samples were sampled from1983 to 2003 at outbreak sites. Human samples used to isolate the virus were sent by physicians as part of routine diagnostics procedures and were verbally authorized (when possible) by patients that were kept anonymous. On the other hand, samples obtained from vertebrates and insects, were provided by veterinarians and entomologists from national health authorities during RVFV outbreaks. No ethical statement for animal experiments was demanded, since no animal experiments were conducted, other than sampling near human cases. Nevertheless, all animal blood sampling was performed while minimizing suffering. In addition, all samples of the study were collected in the course of routine surveillance by national health authorities.

### Virus Isolates

The strains used were obtained from mosquitoes, humans and animals in Senegal and Mauritania. Date of isolation of strains and animal source are listed in Table S1, geographical origins are shown in Figure S2. RVFV samples were obtained from the virus collection of the WHO reference center for arboviruses research in the Institute Pasteur of Dakar. They have been propagated in Vero E6 or AP61 cells cultured in Leibovitz medium L15 supplemented with 5% fetal bovine serum, antibiotics, Fungizone and Tryptose (for AP61 cells). Viruses were harvested after infected cells presented a cytopathic effect. Additional reference sequences from other localities in Africa were obtained from GenBank (http://www.ncbi.nlm.nih.gov/genbank/) and they were listed in Dataset S2.

### Viral RNA extraction and amplification

Viral RNA was extracted from cells culture supernatant by using the QIAgen Viral RNA mini kit (Qiagen, Valencia, CA) according to the manufacturer's instructions and collected in 50 µl of elution buffer. Partial regions of the G2 (718 bp), NSs (601 bp) and L (129 bp) genes were amplified using the sets of primers MRV1a-MRV2g, NS3a-NS2g, and Wag-Xg respectively (Table S3). Reverse transcription-PCR reactions for the cDNA synthesis were performed for NSs and G2 using the AMV Reverse Transcriptase (M5101). Taq DNA polymerase (M1865) of Promega Corporation (Winsconsin, USA) was used for the PCR amplification of both regions. Primers sequences and protocols were previously described [29]. Whereas for the L region the RT-PCR was amplified with the superscript II (SII) (Invitrogen, USA) and Takara taq DNA polymerase (Takara, USA) for the synthesis of the cDNA and polymerase amplification respectively. First strand cDNA synthesis was performed in a final volume of 20 µl by incubating first 10 µl of eluted RNA, 1 µl dNTP (10 mM- Amersham), 1 µl of reverse primer (100 µg/µl) and 1 µl H_2_O at 65°C for 5 min and then rapidly chilled on ice. The following mixture: 4 µl 5xbuffer, 1 µl DTT, 1 µl RNasin Ribonuclease Inhibitor 2500 U (Promega) and 1 µl SII transcriptase (*18080–044*) was added before an incubation at 50°C for 60 min followed by a heat inactivation at 70°C for 15 min. The amplification reaction was initiated with 5 µl of the resulting cDNA as template. Thermocycler program was: one cycle of 95°C for 2 min 30 sec, 45 cycles each one with 95°C for 45 sec, 45°C for 30 sec and 72°C for 45 sec, and one final extension cycle at 72°C for 5 min. RT-PCR amplification from field sample isolates was done in the Institute Pasteur in Dakar, Senegal.

### Sequencing

RT-PCR products were separated by electrophoresis on a 1% agarose gel. Bands of the appropriate molecular size were excised and DNA was recovered using the QiaQuick Gel Extraction Kit (Qiagen) as specified by the manufacturer. Both strands sequencing was performed using the same reverse and forward primers as for the amplification. Sequencing reactions were purified by precipitation: (Hi-Di from Applied Biosystems Foster City, CA) and finally run on an ABI Prism 3100 Genetic Analyzer (Applied Biosystems). DNA sequencing was done at the Microbiology Department of the Biomedical Sciences Institute at the University of São Paulo, Brazil. GenBank accession numbers for S segment sequences are from JN995251 to JN995298 and for M sequences are from JN995299 to JN9954345, while sequences from the L segment portion of the polymerase gene are available from the authors upon request because GenBank requires sequences longer than 200 nucleotides.

### Sequence analysis

Sequences were inspected and assembled with Geneious Pro3.5.4 program (http://www.geneious.com/) and aligned using the multiple sequence alignment algorithm Clustal W [40] and Se-Al v 2.0 (http://tree.bio.ed.ac.uk/software/seal/). To prevent potential biases during phylogenetic inference due to recombination, we first analyzed all sequences with RDP4beta 4.8 program that incorporates RDP, GENECONV, Chimaera, Maxchi, Bootscan, SiScan and 3Seq [41] to uncover evidence for recombination events. Only events with *p*-values≤0.05 that were detected by three or more methods were considered using the Bonferroni correction to prevent false positive results. In addition, intending to infer the selection pressures acting on each genomic segment of RVFV, we estimated the difference between the non-synonymous (*dN*) and synonymous (*dS*) rates per codon site using the Single Likelihood Ancestor Counting (SLAC) algorithm available in HyPhy v0.99 [42], assuming a significance level of 1% (α = 0.01). In the HyPhy output, values of ω are expressed as ω = *dN*-*dS*. Therefore, ω greater than zero are indicative of directional, positive selection (ω>0), while values below zero (ω<0) indicate purifying, negative selection.

### Phylodynamic Analyses

Prior to the analyses, the phylogenetic signal content of the sequence datasets to phylogenetic reconstruction was investigated by Likelihood mapping [43] with TREE-PUZZLE [44]. Phylogenetic trees were generated by Maximum Likelihood (ML) criterion using GARLI v0.96 [45] that uses a stochastic genetic algorithm to estimate simultaneously the best topology, branch lengths and substitution model parameters that maximize the log-Likelihood (lnL). The confidence of ML trees was accessed by the convergence of lnL scores from ten independent replicates. Likewise previous RVFV reports [23], GTR model with Gamma-distributed rate variation (γ) and a proportion of invariable sites (I) substitution model was used. Since we had dates of isolation, we estimated substitution rates per site per year (μ) with R8s v1.71 [46] using the Penalized Likelihood method [47] that employs a semi-parametric approach, using different substitution rates on every branch with a nonparametric roughness penalty, which impose costs according to the model once rates change too quickly from branch to branch. In addition, Maximum Clade Credibility (MCC) trees were inferred using a Markov Chain Monte Carlo (MCMC) Bayesian approach under GTR+γ+I and a relaxed (uncorrelated lognormal) molecular clock [48] with the μ previously estimated on the program BEAST v1.6.1 [49]. Moreover, we investigated the variation in effective population size times the generation time (Ne.g) to infer viral demography by Bayesian Skyride Plots [50]. MCMC convergence was obtained during four independent runs with 50 million of generations, which were sufficient to obtain a proper sample from the posterior at MCMC stationarity. The stationarity of parameters was also assessed by allowing the effective sample sizes (ESS) to reach values above 200 as inspected using Tracer v1.5 (http://tree.bio.ed.ac.uk/software/tracer/). Furthermore, to infer the history of geographical dispersion of RVFV strains, we used a discrete model attributing state characters representing isolation locality of each of the strains with the Bayesian Stochastic Search Variable (BSSVS) algorithm [51] implemented in BEAST v1.6.1. This method estimates the most probable state at each node in the MCC trees, allowing us to reconstruct ancestral positions for ancestral viral lineages along the tree. For phylogeographic reconstructions, each locality was coded as a discrete trait. For Senegal and Mauritania we coded for each locality, down to the city level, while for other African countries we coded localities at national level (coarse grain). We argue that this approach was justified since it allow to focus on the movement within and between Senegal and Mauritania at a finer grain, while also showing its relationship with other African countries at a coarse grain. Likewise, to map routes of spread we resolved cities in Senegal and Mauritania and pinned incoming and outgoing routes at the center of the other African countries.

## Supporting Information

Figure S1 **Maximum clade credibility (MCC) tree summarizing geographical states reconstructions along a time-scaled tree.** Ancestral states reconstructions indicating the most probable location of a lineage in time along colored branches and dates of nodes are shown. Countries are ISO coded as follows: Burkina Faso (BF), Central African Republic (CF), Egypt (EG), Guinea (GN), Kenya (KE), Madagascar (MG), Mauritania (MR), Namibia (NA), Saudi Arabia (SA), Senegal (SN), South Africa (ZA), Tanzania (TZ), Uganda (UG), Zimbabwe (ZW), Angola (AO), Zambia (ZM), Somalia (SO).
(TIF)

Figure S2 **Map from Senegalese and Mauritanian territories.** Circles represent the locations from RVFV isolates.
(TIF)

Table S1 **Source, geographical origin and date of isolations of RVFV strains used in this study.** *Patient died.
(DOC)

Table S2 **Likelihood mapping of the three RVFV genomic segments.** *The percentage of the unresolved quartets is an indicator of phylogenetic suitability from data under analysis. If the percentage is higher, the suitability is lower. Our results suggested that Medium segment is the best for phylogenetic inference due to the lower associated uncertainty.
(DOC)

Table S3 **Description of PCR primers used to amplify Senegal and Mauritania samples.** *Relative position to strain MP12 whose GenBank accession numbers from Small, Medium and Large segments are respectively X53771, M11157 and X56464.
(DOC)

Dataset S1 **Spread of RVFV strains in Africa.** A kml file to picture the history of RVFV migration in Africa along the time, executable in Google earth (http://earth.google.com).
(KML)

Dataset S2 **Additional Reference Sequences of RVFV.** An xls file showing the sequences recovered from GenBank (http://www.ncbi.nlm.nih.gov/genbank/).
(XLS)
