# Supplementary figures and images for: Phylogeography of Rift Valley Fever Virus in Africa Reveals Multiple Introductions in Senegal and Mauritania
Source: PLoS One. 2012 Apr 23;7(4):e35216. doi: 10.1371/journal.pone.0035216 (PMC3335152; doi:10.1371/journal.pone.0035216)

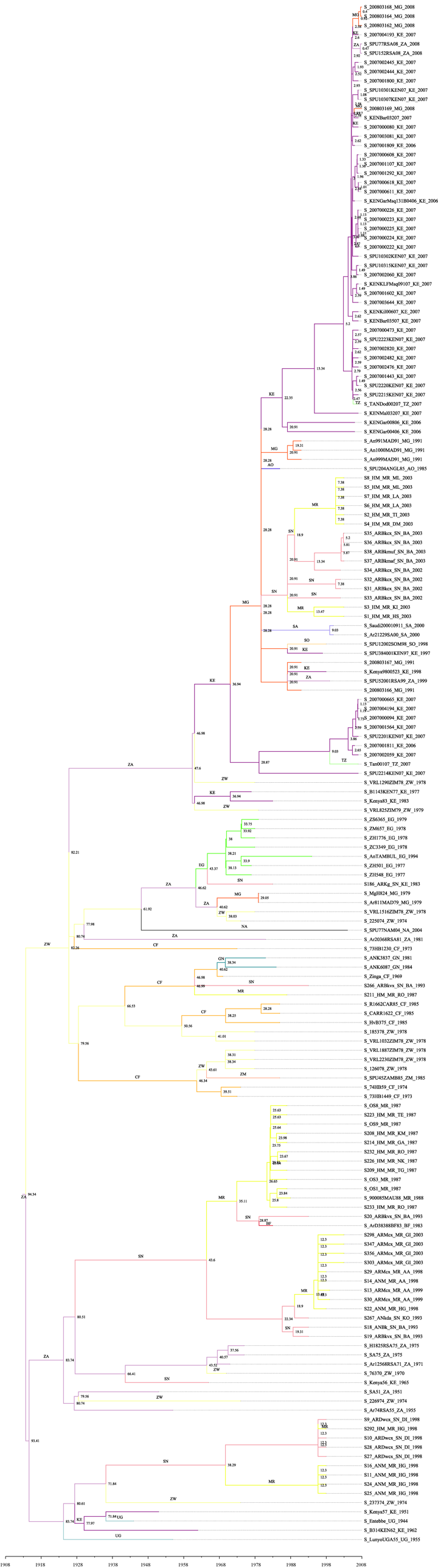

Supplement: Figure S1 — Maximum clade credibility (MCC) tree summarizing geographical states reconstructions along a time-scaled tree. Ancestral states reconstructions indicating the most probable location of a lineage in time along colored branches and dates of nodes are shown. Countries are ISO coded as follows: Burkina Faso (BF), Central African Republic (CF), Egypt (EG), Guinea (GN), Kenya (KE), Madagascar (MG), Mauritania (MR), Namibia (NA), Saudi Arabia (SA), Senegal (SN), South Africa (ZA), Tanzania (TZ), Uganda (UG), Zimbabwe (ZW), Angola (AO), Zambia (ZM), Somalia (SO). (TIF) [file pone.0035216.s001.tif]

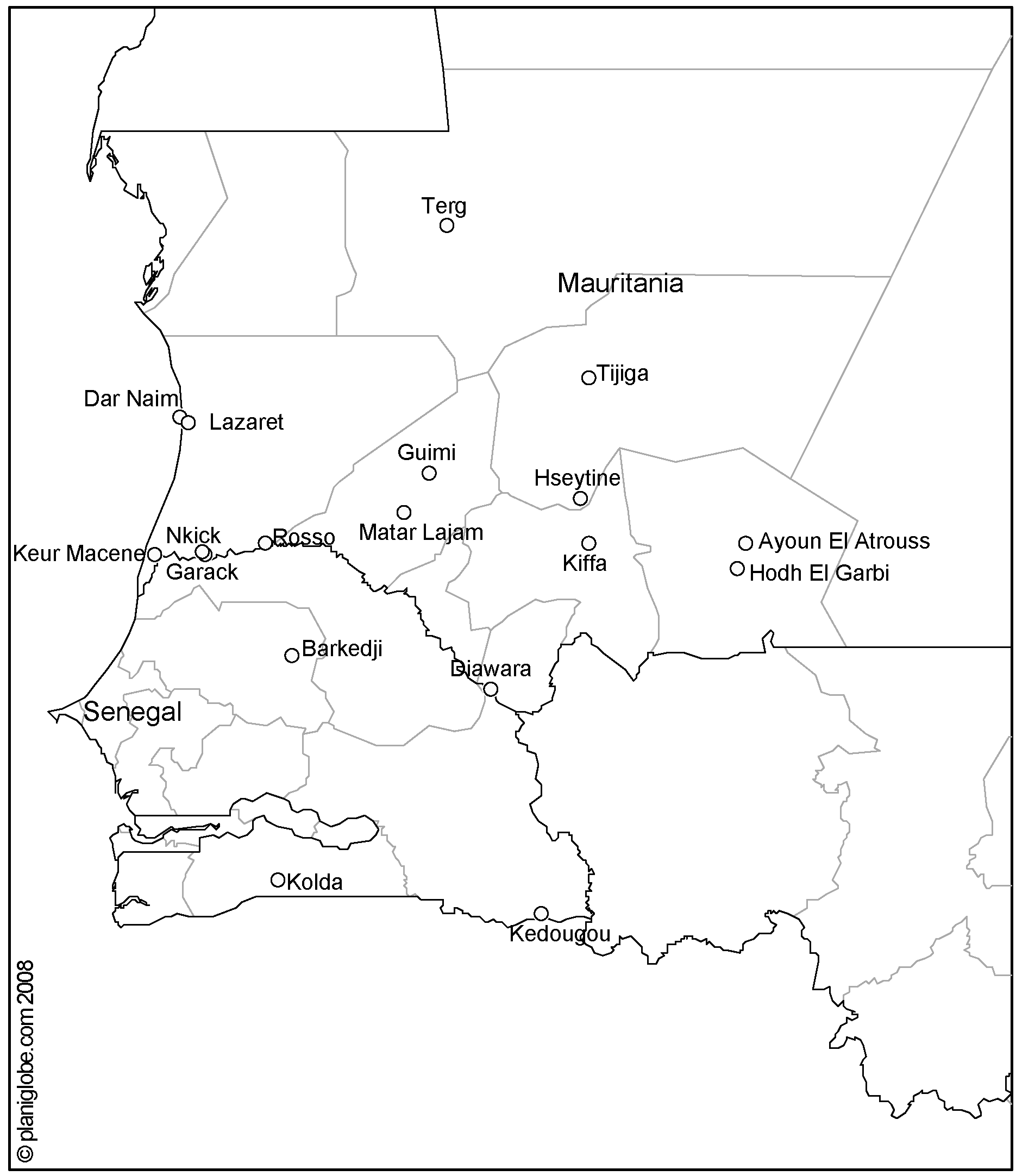

Supplement: Figure S2 — Map from Senegalese and Mauritanian territories. Circles represent the locations from RVFV isolates. (TIF) [file pone.0035216.s002.tif]
