# Supplementary material for: Phylogeography of Rift Valley Fever Virus in Africa Reveals Multiple Introductions in Senegal and Mauritania
Source: PLoS One. 2012 Apr 23;7(4):e35216. doi: 10.1371/journal.pone.0035216 (PMC3335152; doi:10.1371/journal.pone.0035216)
Supplement: Table S1 — Source, geographical origin and date of isolations of RVFV strains used in this study. *Patient died. (DOC) [file pone.0035216.s003.doc]

| Name | Host | Isolation local | Date |
| --- | --- | --- | --- |
| 186-ArDKd83dz | *Aedes dalzieli* | Kédougou (SN) | Oct 1983 |
| 208-HM87 | *Homo sapiens* | K.Macéne (MR) | Oct 1987 |
| 209-HM87 | *Homo sapiens* | Tegmaline (MR) | Oct 1987 |
| 211-HM87 | *Homo sapiens* | Rosso (MR) | Oct 1987 |
| 226-HM87 | *Homo sapiens* | Nkick (MR) | Oct 1987 |
| 214-HM87 | *Homo sapiens* | Garack (MR) | Nov 1987 |
| 223-HM87 | *Homo sapiens* | Terg (MR) | Nov 1987 |
| 232-HM87 | *Homo sapiens* | Rosso (MR) | Nov 1987 |
| 233-HM87 | *Homo sapiens* | Rosso (MR) | Nov 1987 |
| 267-AnDKda93 | *Bos taurus* | Kolda (SN) | Nov 1993 |
| 266-ArDBk93vx | *Aedes vexans* | Barkédji (SN) | Oct 1993 |
| 18-AnDBk93 | *Ovis aries* | Barkédji (SN) | Oct 1993 |
| 19-ArBk93vx | *Aedes vexans* | Barkédji (SN) | Oct 1993 |
| 20-ArBk93vx | *Aedes vexans* | Barkédji (SN) | Oct 1993 |
| 292-HM98 | *Homo sapiens* | Hodh El Garbi (MR) | Oct 1998 |
| 11-ANM98 | *Ovis aries* | Hodh El Garbi (MR) | Oct 1998 |
| 16-ANM98 | *Capra aegragatus* | Hodh El Garbi (MR) | Oct 1998 |
| 22-ANM98 | *Capra aegragatus* | Hodh El Garbi (MR) | Oct 1998 |
| 24-ANM98 | *Ovis aries* | Hodh El Garbi (MR) | Oct 1998 |
| 25-ANM98 | *Ovis aries* | Hodh El Garbi (MR) | Oct 1998 |
| 27-ARDw98cx | *Culex poicilipes* | Diawara (SN) | Nov 1998 |
| 28-ARDw98cx | *Culex poicilipes* | Diawara (SN) | Nov 1998 |
| 9-ARDw98cx | *Culex poicilipes* | Diawara (SN) | Nov 1998 |
| 10-ARDw98cx | *Culex poicilipes* | Diawara (SN) | Nov 1998 |
| 29-ARM99cx | *Culex poicilipes* | Ayoun El Atrouss (MR) | Oct 1999 |
| 13-ARM99cx | *Culex poicilipes* | Ayoun El Atrouss (MR) | Oct 1999 |
| 30-ARM99cx | *Culex poicilipes* | Ayoun El Atrouss (MR) | Nov 1999 |
| 14-ARM99cx | *Culex poicilipes* | Ayoun El Atrouss (MR) | Nov 1999 |
| 34-ARBk02vx | *Aedes vexans* | Barkédji (SN) | Oct 2002 |
| 32-ARBk02cx | *Culex poicilipes* | Barkédji (SN) | Nov 2002 |
| 33-ARBk02cx | *Culex poicilipes* | Barkédji (SN) | Nov 2002 |
| 31-ARBk02cx | *Culex poicilipes* | Barkédji (SN) | Nov 2002 |
| 1-HM03* | *Homo sapiens* | Hseytine (MR) | Oct 2003 |
| 2- HM03 | *Homo sapiens* | Tijiga (MR) | Sep 2003 |
| 3- HM03 | *Homo sapiens* | Kiffa (MR) | Sep 2003 |
| 4- HM03 | *Homo sapiens* | Dar Naim (MR) | Oct 2003 |
| 5- HM03 | *Homo sapiens* | Matar Lajar (MR) | Oct 2003 |
| 6- HM03 | *Homo sapiens* | Lazaret (MR) | Nov 2003 |
| 7- HM03 | *Homo sapiens* | Lazaret (MR) | Nov 2003 |
| 8- HM03* | *Homo sapiens* | Matar Lajar (MR) | Nov 2003 |
| 298-ARM03cx | *Culex poicilipes* | Guimi (MR) | Oct/Nov 2003 |
| 303-ARM03cx | *Culex poicilipes* | Guimi (MR) | Oct/Nov 2003 |
| 347-ARM03cx | *Culex poicilipes* | Guimi (MR) | Oct/Nov 2003 |
| 356-ARM03cx | *Culex poicilipes* | Guimi (MR) | Oct/Nov 2003 |
| 35- ARBk03cx | *Culex poicilipes* | Barkedji (SN) | Oct 2003 |
| 36- ARBk03cx | *Culex poicilipes* | Barkedji (SN) | Oct 2003 |
| 37- ARBk03ma | *Mansonia africana* | Barkedji (SN) | Nov 2003 |
| 38- ARBk03munif | *Mansonia uniformis* | Barkedji (SN) | Oct 2003 |
