# Supplementary material for: Phylogeography of Rift Valley Fever Virus in Africa Reveals Multiple Introductions in Senegal and Mauritania
Source: PLoS One. 2012 Apr 23;7(4):e35216. doi: 10.1371/journal.pone.0035216 (PMC3335152; doi:10.1371/journal.pone.0035216)
Supplement: Table S2 — Likelihood mapping of the three RVFV genomic segments. *The percentage of the unresolved quartets is an indicator of phylogenetic suitability from data under analysis. If the percentage is higher, the suitability is lower. Our results suggested that Medium segment is the best for phylogenetic inference due to the lower associated uncertainty. (DOC) [file pone.0035216.s004.doc]

| **Genetic segment** | **Resolved quartets (%)** | **Unresolved quartets (%)*** | **Partly resolved quartets (%)** |
| --- | --- | --- | --- |
| **Small** | 84.6 | 11.6 | 3.8 |
| **Medium** | 89.6 | 7.5 | 2.9 |
| **Large** | 85.5 | 10.1 | 4.4 |
