# Supplementary material for: Phylogeography of Rift Valley Fever Virus in Africa Reveals Multiple Introductions in Senegal and Mauritania
Source: PLoS One. 2012 Apr 23;7(4):e35216. doi: 10.1371/journal.pone.0035216 (PMC3335152; doi:10.1371/journal.pone.0035216)
Supplement: Table S3 — Description of PCR primers used to amplify Senegal and Mauritania samples. *Relative position to strain MP12 whose GenBank accession numbers from Small, Medium and Large segments are respectively X53771, M11157 and X56464. (DOC) [file pone.0035216.s005.doc]

| **Genomic segment** | **Position*** | **Primer sequence** |
| --- | --- | --- |
| **Small** | 729 - 710 | ATGCTGGGAAGTGATGAGCG |
| **Small** | 61 - 80 | TGATTTGCAGAGTGGTCGTC |
| **Medium** | 772-790 | CAAATGACTACCAGTCAGC |
| **Medium** | 1580-1563 | GGTGGAAGGACTCTGCGA |
| **Medium** | 1342-1326 | CCTGACCCATTAGCATG |
| **Large** | 4440-4457 | ATTCTTATTCCCGAATAT |
| **Large** | 4651-4634 | TTGTTTTGCCTATCCTAC |
